# Poor health and refraining from seeking healthcare are associated with comprehensive health literacy among refugees: a Swedish cross-sectional study

**DOI:** 10.1007/s00038-017-1074-2

**Published:** 2018-02-26

**Authors:** Josefin Wångdahl, Per Lytsy, Lena Mårtensson, Ragnar Westerling

**Affiliations:** 1 Department of Public Health and Caring Science, Uppsala University, Uppsala, Sweden; 2 Institution of Department of Neuroscience and Physiology/Occupational Therapy, University of Gothenburg, Gothenburg, Sweden

**Keywords:** Health literacy, General health, Psychological well-being, Refrained from seeking healthcare, Healthcare, Refugees

## Abstract

**Objectives:**

The objectives of the study were to explore the distributions of comprehensive health literacy (CHL), general health, psychological well-being, and having refrained from seeking healthcare among refugees in Sweden. Further objectives were to examine associations between CHL and the above-mentioned factors.

**Methods:**

A cross-sectional study was conducted among 513 refugees speaking Arabic, Dari, and Somali. Participants in the civic orientation course in Sweden responded to a questionnaire. CHL was measured using the HLS-EU-Q16 questionnaire. Uni- and multivariate logistic regression was used to investigate potential associations.

**Results:**

The majority of the respondents had limited CHL, and about four of ten had reported poor health and/or having refrained from seeking healthcare. Limited CHL was associated with having reported poor health and having refrained from seeking healthcare.

**Conclusions:**

A considerable proportion of the refugees in Sweden have limited CHL, and report less than good health and impaired well-being, or that they have refrained from seeking healthcare. Furthermore, CHL is associated with the above-mentioned factors. Efforts are needed to promote refugees’ CHL, optimal health-seeking behavior, and health.

## Introduction

There were about 24.5 million asylum seekers and refugees worldwide in 2015, most coming from Syria, Afghanistan, and Somalia (UNHCR 2015). The third largest recipient country of individual applications for asylum the same year was Sweden.

Many refugees have poor self-perceived general health (Lecerof et al. 2017; Nielsen and Krasnik 2010) and psychological well-being (Lecerof et al. 2017; Tinghög et al. 2016; Yaser et al. 2016), and refugees’ health status often deteriorates over time (Thomas 2016). In addition, many refugees refrain from seeking healthcare and from participating in health promoting and disease preventing activities (Ingleby 2012; Lecerof et al. 2017).

Poor knowledge of the healthcare service and how to use it, difficulties adapting to a new healthcare system, and language barriers are all well-known hinders to healthcare for new migrants (Hadgkiss and Renzaho 2014) but are also all highly related to health literacy (HL). Migrants’ limited HL and a failure of the organizations providing health information to meet this limitation (Gele et al. 2016; Kimbrough Benneth 2007) could, therefore, be of importance for refugees’ health and healthcare utilization.

Many different definitions of HL are given in the literature (Sorensen et al. 2012), most having a medical approach that focuses mainly on individuals’ ability to read information and instructions about health, i.e., functional HL (Nutbeam 2008). In our study, we focus on comprehensive HL (CHL) as defined by Sorensen et al. (2012), which is based on a public health perspective. According to this, HL is something that “links to literacy and entails people’s knowledge, motivation and competences to access, understand, appraise, and apply health information in order to make judgments and decisions in everyday life concerning healthcare, disease prevention and health promotion to maintain or improve quality of life during the life course”.

An instrument has been developed and validated to measure CHL specifically (Pelikan and Ganahl 2017; Sorensen et al. 2013). Limited CHL has been found to be influenced by socio-demographic factors such as old age, low education, and poor economy (Levin-Zamir et al. 2016; Palumbo et al. 2016; Sorensen et al. 2015). In addition, CHL has been found to be associated with various health outcomes, for example, with poor general health (Levin-Zamir et al. 2016; Palumbo et al. 2016; Sorensen et al. 2015), having a long-term illness, high use of healthcare services (Sorensen et al. 2015), and gaining fewer benefits from disease preventing efforts (Wångdahl et al. 2015). In comparison with the general population, migrants and refugees often have more limited CHL (Gele et al. 2016; Wångdahl et al. 2014). However, most HL research has focused on functional HL, and there is a lack of CHL research that focuses on refugees.

The influx of refugees in many countries is high and refugees are not a homogenous group (Thomas 2016); more knowledge about the health status and health-seeking behaviors in different sub-groups in different contexts is needed. In addition, there is no knowledge of associations between CHL, general health, psychological well-being, and having refrained from seeking healthcare among refugees. Information about this may contribute to the understanding of inequality in health and healthcare, and indicate whether it is important to take refugees’ CHL into account in healthcare and in health promoting activities during the establishment period.

### Objectives

The objectives of this study were to explore the distributions of CHL, general health, psychological well-being, and having refrained from seeking healthcare among refugees in Sweden. Further objectives were to examine associations between CHL and the above-mentioned factors.

## Methods

### Study design and setting

The study has a cross-sectional design. Data were collected during January–July 2015 among refugees participating in courses in civic orientation in the largest city in Sweden. According to Swedish law, all refugees with a residence permit newer than 2 years shall be offered 60 h of civic orientation, i.e., information about Swedish society. The study was approved by the Ethical Committee of Uppsala, Sweden, registration number 2014:526.

### Study population and data collection

A consecutive selection (Roach 2001) was used, i.e., all course participants fulfilling the inclusion criteria during the study period were selected. Inclusion criteria were: speaking Arabic, Dari or Somali, and being present the first day of the course in civic orientation.

Information about the study and that participation was voluntary was given in writing in the refugees’ native language. On the first day of the course, a researcher and a language supporter visited each group to collect data. Participants consenting to participate were given a questionnaire, described below, which they completed on site. Those with limited reading and writing skills were assisted by the language supporter. Altogether, 44 groups with between 5 and 20 participants were visited. On average, less than one participant per group declined to take part. A total of 513 refugees answered the questionnaire.

### Measures

The questionnaire contained 64 questions. The primary purpose was to map the characteristics of and the need for health information among the participants in the civic orientation at startup. The questions were translated into Arabic, Dari, and Somali following guidelines for the translation of instruments (Guillemin et al. 1993), i.e., translated, back-translated, and tested through a cognitive interview with three-to-four refugees in each language. Some items were reused from the previous studies with similar study populations (Wångdahl et al. 2014, 2015). For the purpose of this study, only questions regarding health status, having refrained from seeking healthcare, CHL, demography, and migration-related factors, were analyzed.

### Variables

#### Dependent variables

All dependent variables were self-reported.

General health was measured by the question “How do you assess your overall health status?” (very good, good, fair, poor, and very poor), which has been widely used in research (Nielsen and Krasnik 2010). It measures physical, social, and emotional health, and aims to measure health in general rather than temporary health problems. The variable was dichotomized for analytic purposes into good (good and very good) and less than good (fair, poor, and very poor) health, in line with the previous studies (Carneiro et al. 2013; Chandola and Jenkinson 2000).

Psychological well-being was measured by the shortest form of the General Health Questionnaire (GHQ12) (Goldberg et al. 1997). It consists of statements about the respondents’ state during the past few weeks, asking them to rate how much it deviates from their usual state. Worldwide, in various cultures, it is used for research purposes and as a screening tool for mental health problems. Its validity is judged to be satisfactory (Bowe 2017; Goldberg et al. 1997). A sum score (0–12) was calculated by the usual method (Goldberg et al. 1997), and thereafter dichotomized into two categories: impaired psychological well-being (≤ 2 points) and not impaired psychological well-being (≥ 3 points). This threshold has been used in the previous studies to distinguish persons with impaired psychological well-being from others (Goldberg et al. 1997; The Public Health Agency of Sweden 2014).

Having refrained from seeking healthcare was measured by the question “In the past three months, have you felt a need for help with health problems, but have not sought healthcare?” (yes; no). Follow-up questions were asked about the reason for having refrained from seeking healthcare. The question has been used in the previous studies (Lecerof et al. 2017; Wamala et al. 2007) and in the Swedish public health survey, as an indicator for identifying possible needs for action in healthcare (The Public Health Agency of Sweden 2014).

### Independent variable

CHL was measured using the Swedish version of the short European HL questionnaire (HLS-EU-Q16) (Pelikan and Ganahl 2017), a self-reporting instrument consisting of 16 items measuring four dimensions of HL as defined by Sorensen et al. (Sorensen et al. 2012). The instrument is validated in the general population as well as among migrants in Europe (Pelikan and Ganahl 2017). It is one of the few instruments trying to capture several HL dimensions. All items and response alternatives are presented in “Appendix 1”. The response alternative “don’t know” was not used, since it had in the previous studies (Wångdahl et al. 2014, 2015) resulted in many missing values for the overall sum score of CHL. In the original, oral, version, “don’t know” is used only if the respondent does not answer any of the four given response alternatives. An overall sum score of the response values was calculated and divided into three CHL categories: inadequate (0–8), problematic (9–12), and sufficient (13–16). The same categories and cut-off values were used by the instrument’s developers (Pelikan and Ganahl 2017).

#### Potential confounders

Potential confounders analyzed in the study can be divided into three groups: demographic data, migration-related data, and long-term illness.

Demographic data used were country of birth, sex, age and education level. During the analysis, age and education level groups were created using the same intervals as in the previous studies (Wångdahl et al. 2014, 2015).

Migration-related data used were years with and reasons for residence permit in Sweden, and having participated in a health examination for asylum seekers (HEA). Years with a residence permit were measured on a continuous scale but divided into “less than one year” and “one year or more” in the analysis. Having participated in an HEA was measured with the question “Have you been offered a health examination for asylum seekers free of charge?” (yes, and I did participate; yes, but I didn’t participate; no; I don’t know), which has been used previously (Wångdahl et al. 2015). In the analyses, the answer categories were dichotomized into having participated/or not in the HEA. Those responding ‘I don't know’ responses were treated as missing values.

Long-term illness was measured by the question “Do you have any long-term illnesses, problems after an accident, any functional difficulties, or other long-term health problems?” (yes; no). The question is included in the Minimum European Health Module (Eurostat Statistics Explained 2016) and is frequently used in research (Lecerof et al. 2017; Sorensen et al. 2015).

### Statistical analysis

Chi-square tests were used to investigate differences in general health, psychological well-being, and having refrained from seeking healthcare between groups with different CHL levels.

Uni- and multivariate logistic regression was used to investigate the associations between *CHL,* the dependent variables of general health status, psychological well-being, and having refrained from seeking healthcare, adjusting for potential confounders. Variables were inserted stepwise, manually, model wise. In the first models, *CHL* and demographic variables were included as independent variables. The second models included the same variables as in model 1 plus migration-related variables. The third model included the same variables as model 2 plus long-term illness. The results are presented as crude and adjusted odds ratios (OR) with 95% confidence intervals (CI) and *p* values. A *p* value of < 0.05 was considered statistically significant, and all analyses were two-sided. The Statistical Package for the Social Sciences (SPSS) version 24 (Chicago, IL, USA) was used for all statistical analyses.

## Results

### Characteristics of the study population

The majority of the respondents had limited (inadequate or problematic) CHL (67%). Most of the respondents were born in Syria (81%) and most of the respondents were men (80%). The mean age was 38 (range 18–74), and the most common length of education was 12 years or more (39%). Almost everyone had had a residence permit in Sweden for a period of 2 years or less (96%) and had received it for reasons of asylum (76%). Most had participated in an HEA (70%) and about one-third reported a long-term illness (27%). Distributions of study population characteristics are shown in Table 1.

**Table 1:** Demographic- and migratory related characteristics, long-term illness, and comprehensive health literacy among newly arrived refugees, Sweden 2015

Variables	Total (*n* = 513)	
	*N*	(%)^a^
CHL^b^ (*n* = 494)		
Inadequate	190	38.5
Problematic	143	28.9
Sufficient	161	32.6
Country of birth (*n* = 495)		
Afghanistan	19	3.8
Iraq	22	4.4
Iran	16	3.2
Somalia	13	2.6
Syria	401	81.0
Other	24	4.8
Sex (*n* = 503)		
Man	391	77.7
Woman	112	22.3
Age (*n* = 465)		
18–24	65	14.0
25–44	265	57.0
45 years or older	135	29.0
Education (*n* = 507)		
None	22	4.3
1–6 years	63	12.4
7–9 years	88	17.4
10–12 years	136	26.8
More than 12 years	198	39.1
Years of residential permit (*n* = 445)		
Less than 1 year	188	42.2
1–2 year	237	53.3
More than 2 years	20	4.5
Reasons for residential permit (*n* = 501)		
Asylum	381	76.0
Family reunion	116	23.2
Other reason	4	0.8
Having participated in HEA^c^ (*n* = 487)		
Yes	342	70.2
No	145	29.8
Long-term illness (*n* = 498)		
No	363	72.9
Yes	135	27.1

^a^Distribution %

^b^Comprehensive health literacy

^c^Health examination for asylum seekers

### General health

Forty-one percent estimated their overall general health as less than good. Chi-square tests indicated associations between health and CHL (Table 2): the lower the CHL, the poorer the general health. The univariate analysis (Table 3) showed that those with inadequate CHL were 2.75 times as prone, and those with problematic CHL 1.89 times as prone, to report less than good health as those with sufficient CHL. When adjusting for demographic and migration-related factors and self-reported long-term illness in the multivariate analysis, the association remained significant and the OR did not change remarkably. Other factors associated with poor self-reported health in the full model were high age, low education level, and having a long-term illness (Table 3).

**Table 2:** Distribution of general health, psychological well-being, and having refrained from seeking healthcare, among newly arrived refugees, and in sub-groups with different comprehensive health literacy levels, Sweden 2015

CHL^a^					
Variables	Total (*N* = 513), *N* (%)^b^	Inadequate *n* (%)^b^	Problematic *n* (%)^b^	Sufficient *n* (%)^b^	*p* value^c^
General health (*n* = 508)					< 0.001
Good or very good health	302 (59.4)	93 (49.2)	83 (58.5)	117 (72.7)	
Less than good health	206 (40.6)	96 (50.8)	59 (41.5)	44 (27.3)	
Psychological well-being (*n* = 511)					< 0.001
Good psychological well-being	330 (64.6)	94 (49.5)	91 (63.6)	132 (82.0)	
Impaired psychological well-being	181 (35.4)	96 (50.5)	52 (36.4)	29 (18.0)	
Having refrained from seeking healthcare (*n* = 497)					0.002
No	317 (63.8)	107 (58.5)	83 (58.9)	118 (75.2)	
Yes	180 (36.2)	76 (41.5)	58 (41.1)	39 (24.8)	

^a^Comprehensive health literacy

^b^Distribution %

^c^*p* values for differences obtained using Chi-square statistics

**Table 3:** Odds ratio for having less than good health among newly arrived refugees, Sweden 2015

Variables	Crude OR (95% CI)	Model 1 OR^a^ (95% CI)	Model 2 OR^b^ (95% CI)	Model 3 OR^c^ (95% CI)
CHL^d^				
Sufficient	1	1	1	1
Problematic	1.89 (1.17–3.06)*	2.10 (1.22–3.62)**	2.21 (1.22–4.02)**	1.96 (1.02–3.77)*
Inadequate	2.75 (1.75–4.30)***	2.38 (1.43–3.95)**	2.72 (1.55–4.77)***	2.93 (1.58–5.42)**
Sex				
Man	1	1	1	1
Woman	1.43 (0.94–2.18)	1.33 (0.80–2.19)	1.47 (0.84–2.58)	1.63 (0.89–2.99)
Age				
18–24 years	1	1	1	1
25–44 years	2.55 (1.27–5.13)**	2.82 (1.35–5.92)**	3.63 (1.69–8.27)**	2.74 (1.16–6.46)*
45 years or older	8.98 (4.29–18.82)***	9.17 (4.17–20.14)***	11.42 (4.62–28.20)***	5.26 (2.02–13.69)**
Education level				
More than 12 years	1	1	1	1
7–12 years	1.16 (0.78–1.73)	1.22 (0.77–1.92)	1.11 (0.67–1.83)	1.23 (0.71–2.12)
0–6 years	2.62 (1.55–4.42)***	1.84 (0.99–3.43)	2.31 (1.14–4.68)*	2.20 (1.01–4.77)*
Years with residential permit				
Less than 1 year	1		1	1
1 year or more	1.22 (0.83–1.79)		0.92 (0.57–1.48)	0.81 (0.49–1.35)
Reasons for residential permit				
Asylum	1		1	1
Family reunion or other	1.18 (0.78–1.79)		0.63 (0.33–1.21)	0.65 (0.33–1.31)
Having participated in a HEA^e^				
Yes	1		1	1
No	1.05 (0.70–1.56)		0.90 (0.51–1.60)	1.16 (0.62–2.15)
Long-term illness				
No	1			1
Yes	8.35 (5.28–13.20)***			6.93 (3.87–12.41)***

*95% CI* 95% confidence interval

*Statistically significant *p* value < 0.05; **Statistically significant *p* value < 0.01; ***Statistical *p* value < 0.001

^a^Adjusted for comprehensive health literacy, sex, age, and education level

^b^Adjusted for CHL, sex, age, education level, years with residential permit, reasons for residential permit, and having participated in a HEA

^c^Adjusted for CHL, sex, age, education level, years with residential permit, reasons for residential permit, having participated in HEA, and long-term illness

^d^Comprehensive health literacy

^e^Health examination for asylum seekers

### Psychological well-being

Just over a third of the respondents were classified as having impaired psychological well-being (35%), and Chi-square tests indicated associations between psychological well-being and CHL (Table 2). The lower the CHL, the greater the number of respondents that were classified as having impaired psychological well-being. The univariate analysis (Table 4) showed that those with inadequate CHL were 4.65 times as prone, and those with problematic CHL 2.60 times as prone, to have impaired psychological well-being as those with sufficient CHL. When adjusting for demographic and migrant-related factors and self-reported long-term illness in the multivariate analysis, the association remained significant and the OR did not change remarkably.

**Table 4:** Odds ratio for having impaired psychological well-being among newly arrived refugees, Sweden 2015

Variables	Crude OR (95% CI)	Model 1 OR^a^ (95% CI)	Model 2 OR^b^ (95% CI)	Model 3 OR^c^ (95% CI)
CHL^d^				
Sufficient	1	1	1	1
Problematic	2.60 (1.54–4.41)***	2.64 (1.50–4.63)**	2.37 (1.29–4.34)**	2.35 (1.27–4.33)**
Inadequate	4.65 (2.84–7.61)***	4.91 2.93–8.40)***	4.90 (2.79–8.76)***	4.86 (2.74–8.66)***
Sex				
Man	1	1	1	1
Woman	1.06 (0.69–1.65)	1.16 (0.71–1.90)	0.92 (0.53–1.62)	0.94 (0.53–1.66)
Age				
18–24 years	1	1	1	1
25–44 years	1.18 (0.66–2.10)	1.10 (0.59–2.05)	0.87 (0.45–1.67)	0.80 (0.41–1.56)
45 years or older	1.27 (0.68–2.38)	1.19 (0.60–2.34)	0.87 (0.41–1.82)	0.71 (0.32–1.57)
Education				
More than 12 years	1	1	1	1
7–12 years	1.01 (0.67–1.51)	0.99 (0.63–1.55)	1.00 (0.61–1.64)	1.05 (0.64–1.73)
0–6 years	1.82 (1.08–3.06)*	1.37 (0.75–2.52)	1.45 (0.72–2.90)	1.43 (0.71–2.88)
Years with residential permit				
Less than 1 year	1		1	1
1 year or more	1.06 (0.71–1.58)		1.08 (0.68–1.71)	1.06 (0.66–1.69)
Reasons for residential permit				
Asylum	1		1	1
Family reunion or other	1.46 (0.99–2.21)		0.98 (0.52–1.83)	1.04 (0.55–1.95)
Having participated in a HEA^e^				
Yes	1		1	1
No	1.58 (1.06–2.35)*		1.65 (0.94–2.86)	1.69 (0.96–2.98)
Long-term illness				
No	1			1
Yes	1.30 (0.87–1.96)			1.42 (0.82–2.46)

*95% CI* 95% confidence interval

*Statistically significant *p* value < 0.05; **Statistically significant *p* value < 0.01; ***Statistical *p* value < 0.001

^a^Adjusted for comprehensive health literacy, sex, age, and education level

^b^Adjusted for CHL, sex, age, education level, years with residential permit, reasons for residential permit, and having participated in HEA

^c^Adjusted for CHL, sex, age, education level, years with residential permit, reasons for residential permit, having participated in HEA, and long-term illness

^d^Comprehensive health literacy

^e^Health examination for asylum seekers

### Having refrained from seeking healthcare

Just over a third (36%) reported having refrained from seeking healthcare in the most recent 3 months. The most common reasons for this were: language problems (40%), did not think that help could be obtained (24%), would wait for a while (19%), and did not know where to go (19%) (not presented in any table). The Chi-square tests indicated associations between having reported refraining from healthcare and CHL (Table 2); the lower the CHL, the more respondents reported having refrained from seeking healthcare. The univariate analysis (Table 5) showed that those with inadequate CHL were 2.15 times as prone, and those with problematic CHL 2.11 times as prone, to having refrained from healthcare as those with sufficient CHL. When adjusting for demographic and migrant-related factors and self-reported long-term illness in the multivariate analysis (Table 5), the association remained significant and the OR did not change remarkably. Other factors associated with having refrained from seeking healthcare in the full model were high education level, not having participated in the HEA, and having a long-term illness (Table 5).

**Table 5:** Odds ratio for having refrained from seeking healthcare among newly arrived refugees, Sweden 2015

Variables	Crude OR (95% CI)	Model 1 OR^a^ (95% CI)	Model 2 OR^b^ (95% CI)	Model 3 OR^c^ (95% CI)
CHL^d^				
Sufficient	1	1	1	1
Problematic	2.11 (1.29–3.46)**	2.05 (1.22–3.46)**	1.96 (1.11–3.44)*	1.75 (0.97–3.15)
Inadequate	2.15 (1.35–3.43)**	2.27 (1.35–3.67)**	2.22 (1.29–3.84)**	2.12 (1.20–3.73)**
Sex				
Man	1	1	1	1
Woman	1.37 (0.89–2.11)	1.40 (0.87–2.26)	1.45 (0.85–2.49)	1.58 (0.90–2.77)
Age				
18–24 years	1	1	1	1
25–44 years	1.76 (0.97–3.20)	1.73 (0.93–3.25)	1.58 (0.83–3.02)	1.26 (0.65–2.47)
45 years or older	1.31 (0.68–2.52)	1.31 (0.66–2.62)	1.20 (0.56–2.55)	0.60 (0.26–1.37)
Education				
0–6 years	1	1	1	1
7–12 years	1.28 (0.74–2.21)	1.53 (0.82–2.87)	1.88 (0.89–3.95)	2.38 (1.09–5.21)*
12 years or more	1.48 (0.85–2.57)	1.64 (0.87–3.08)	2.08 (0.99–4.37)	2.44 (1.11–5.36)*
Years with residential permit				
Less than 1 year	1		1	1
1 year or more	1.07 (0.72–1.60)		1.16 (0.73–1.83)	1.09 (0.68–1.76)
Reasons for residential permit				
Asylum	1		1	1
Family reunion or other	0.97 (0.62–1.50)		0.56 (80.29–1.07)	0.59 (0.02–1.14)
Having participated in a HEA^e^				
Yes	1		1	1
No	1.52 (1.02–2.29)*		1.97 (1.14–3.42)*	2.45 (1.37–4.37)**
Long-term illness				
No	1			1
Yes	2.52 (1.67–3.80)***			3.96 (2.25–6.98)***

*95% CI* 95% Confidence interval

*Statistically significant *p* value < 0.05; **Statistically significant *p* value < 0.01; ***Statistically *p* value < 0.001

^a^Adjusted for comprehensive health literacy, sex, age, and education level

^b^Adjusted for CHL, sex, age, education level, years with residential permit, reasons for residential permit, and having participated in HEA

^c^Adjusted for CHL, sex, age, education level, years with residential permit, reasons for residential permit, having participated in HEA, and long-term illness

^d^Comprehensive health literacy

^e^Health examination for asylum seekers

## Discussion

The main findings are that a considerable proportion of the refugees have limited CHL, have reported poor health or that they have refrained from seeking healthcare, and that limited CHL was associated with both having reported poor health and having refrained from seeking healthcare.

Because of the cross-sectional design of the study, it is not possible to draw causal conclusions. However, according what limited CHL means, i.e., having difficulties with accessing, understanding, appraising, and applying health information (Sorensen et al. 2012), and to pathway models showing how HL affects health through health behaviors (Rowlands et al. 2017; Sorensen et al. 2012; Sun et al. 2013), it is likely that limited CHL at least contributes to refraining from seeking healthcare and to poor health among refugees.

From a theoretical perspective, the large proportion of limited CHL among refugees is understandable. Arguably, it is difficult to access, understand, appraise, and apply health information in a new country where you do not fully master the language or system, and do not have a social network that can help you. The large proportion of refugees with limited CHL is in line with the previous results that have examined CHL levels among migrants (Gele et al. 2016; Ng and Omariba 2014; Wångdahl et al. 2014). However, in Gele’s (Gele et al. 2016) and Ng’s (Ng and Omariba 2014) studies, it is not clear whether study populations were refugees. In addition, these populations had, on average, been in the country longer than the refugees in our study. The only earlier study with a study population similar to ours had the many missing CHL values (Wångdahl et al. 2014). The findings in this study thus support the notion that limited CHL is common among newly arrived refugees.

High proportions of refugees reporting less than good general health and/or impaired psychological well-being have also been reported previously (Nielsen and Krasnik 2010; Tinghög et al. 2016; Yaser et al. 2016). The results are notable as they indicate that about 40% of the refugees are at increased risk of further health problems, as self-perceived health is associated with sick leave (Carneiro et al. 2013), mortality, and morbidity (Chandola and Jenkinson 2000). The high proportion of refugees having reported that they have refrained from seeking healthcare is consistent with, as far as we know, the only previous study examining this among refugees (Lecerof et al. 2017). However, as our study included refugees from other countries of origin than the previous study, the results imply validity for other groups of refugees as well.

That limited CHL is associated with poor self-perceived health has been shown before (Levin-Zamir et al. 2016; Palumbo et al. 2016; Sorensen et al. 2015). However, those studies did not focus on refugees. That it is more common among refugees with limited CHL to have impaired psychological well-being is new knowledge as well. As far as we know, this association has not been examined either among refugees or in any general population before. Based on the fact that CHL is not static (Sorensen et al. 2012) and pathway models that show that CHL affect health behaviors and health outcomes (Rowlands et al. 2017; Sorensen et al. 2012; Sun et al. 2013), our interpretation is that limited CHL can be one of the several explanatory factors for the high ill health of refugees.

That limited CHL is more common among those having reported that they have refrained from seeking healthcare is new knowledge, as well. One reason can be that refugees with limited CHL receive less information about their right to health or how to access healthcare when they participate in the health examination for asylum seekers (Wångdahl et al. 2015). A further factor is that those with limited CHL experience that they receive less help with their health problems during that examination, which, according to other research, is critical, because dissatisfaction with healthcare and unmet health needs could lead to an avoidance of seeking care in the future (Jonzon et al. 2015; Suphanchaimat et al. 2015). The refugees’ own explanations why they refrained from seeking healthcare indicate that healthcare utilization was suboptimal. It also indicates that the healthcare does not meet the needs of refugees with limited CHL.

However, in comparison with research showing associations between limited CHL, many visits to emergency departments, and high use of healthcare services in general populations (Palumbo et al. 2016), our results showing an association between limited CHL and having refrained from seeking healthcare were not quite expected. The different results may be due to different study populations. Participants in the previous studies were from the general population, while participants in the present study were newly arrived refugees. It is, therefore, reasonable that the refugees in our study to a larger extent than individuals in general have refrained from seeking healthcare, due to language problems and lack of knowledge of where to go. These were also the reasons mentioned by the participants themselves.

Owing to the high influx of refugees worldwide (UNHCR 2015), the results in our study are worrying and could be interpreted as a potential public health concern. The results also indicate that some ill health present in newly arrived refugees might be unnecessary and should be able to be reduced if efforts are made to promote and take into account limited CHL. One way of acting upon the limited CHL is for organizations working with refugees to become more health literate, i.e., to be able to make it easier for refugees to navigate, understand, and use information and services enabling them to take care of their own health (Brach et al. 2012). However, refugees' own health competences, which might differ from common Western clinical praxis, may not necessarily be inappropriate. This should be taken into consideration to reduce the risk of paternalism and assimilation (Ingleby 2012). According to recommendations from WHO Europe (2013), we also suggest an increased use of cultural mediators who can communicate with refugees in a linguistic and culturally appropriate manner. However, additional quantitative and qualitative research is needed to further examine how effective and appropriate those methods are. Furthermore, CHL instruments specifically targeting refugees and migrants are needed, as is research that investigates causal linkages between CHL, different health behaviors, and health outcomes. In addition, studies investigating the reasons why refugees refrain from seeking healthcare are needed.

### Strengths and limitations

The strengths of the study include the high response rate and that the study population includes refugees with both high and low education levels, representing the actual situation.

However, the study also has limitations that should be kept in mind when considering the results. One is that CHL is used as an independent variable. According to the integrated model for HL (Sorensen et al. 2012), CHL can also be a result of poor health, i.e., may be both an independent and a dependent factor. Another limitation is that the translations of the HLS-EU-Q16 used to measure CHL have only been tested for face validity in the target group for this study. However, its validity has been tested more extensively in several other languages, including among migrants (Pelikan and Ganahl 2017). Another concern is the items in HLS-EU-Q16 in which the respondent should judge how difficult they experience different health-related situations to be. Answering them can be difficult if you have not experienced them, and some respondents might have responded to how they experienced the situation at home, instead of in the new country. That the HLS-EU-Q16 is based on self-assessments could have influenced the estimations of CHL, as those with higher CHL levels might have been more self-critical and assessed their ability to handle different situations as being lower and thus would score lower on the scale.

Another limitation in the use of self-reporting measurements in the study is that views on health and healthcare can vary with the country of birth and its culture (Ingleby 2012; Suphanchaimat et al. 2015) and thus could influence interpretations of and answers to health-related questions. However, the measures used for self-reported general health, psychological well-being, and long-term illness are all three established instruments and indicators used in research and public health surveys in many different countries and cultures (Eurostat Statistics Explained 2016; Goldberg et al. 1997; Lecerof et al. 2017; Nielsen and Krasnik 2010; Sorensen et al. 2015). Different views of health and health-seeking behaviors could also have affected the interpretations of and answers to the question about having refrained from seeking healthcare. The type of health problem, its seriousness, or what kind of healthcare was not searched for are not specified, which makes it difficult to examine this in more depth. In addition, the response to that question may depend on the state of health of the individual. However, the results show that having refrained from seeking healthcare was more common among refugees with a long-term illness (Table 5), indicating that a large proportion of refugees with health problems actually has refrained from seeking healthcare.

The threshold used for impaired psychological well-being and the use of the same threshold for the whole study population could be questioned, as the respondents come from countries with different cultural backgrounds. However, we used an established threshold used in several previous studies (Goldberg et al. 1997; The Public Health Agency of Sweden 2014). If we had used an alternative recommended threshold, based on the mean of the GHQ-12 score in the study population (Goldberg et al. 1998), the threshold score should have been the same (based on a mean score of 2.63).

### Conclusion

A considerable proportion of the refugees in Sweden have limited CHL and report less than good health and impaired well-being, or that they have refrained from seeking healthcare. Moreover, limited CHL is associated with reporting less than good health, having impaired psychological well-being, and having refrained from seeking healthcare. Efforts are needed to promote refugees’ CHL, optimal health-seeking behavior, and health.

## Appendix 1 Modified version of the HLS-EU-Q16

Developed by J. Wångdahl och L. Mårtensson based on the original version, HLS-EU Consortium (2012).

**Table:** **Questions about health information**

Questions about how it is for you to find, understand, and use information related to health, illness, and medical care				
*Select the option on each line that best matches your answer*				
	Very easy	Easy	Difficult	Very difficult
a. How easy/difficult is it for you to find information on treatments of illnesses that concern you?	□	□	□	□
b. How easy/difficult is it for you to find out where to get professional help when you are ill (e.g., doctor, pharmacist, or psychologist)?	□	□	□	□
c. How easy/difficult is it for you to understand what your doctor says to you?	□	□	□	□
d. How easy/difficult is it for you to understand your doctor’s or pharmacist’s instructions on how to take a prescribed medicine?	□	□	□	□
e. How easy/difficult is it for you to judge when you need to get a second opinion from another doctor?	□	□	□	□
f. How easy/difficult is it for you to use information the doctor gives you to make decisions about your illness?	□	□	□	□
g. How easy/difficult is it for you to follow instructions from your doctor or pharmacist?	□	□	□	□
h. How easy/difficult is it for you to find information on how to manage mental health problems such as stress and depression?	□	□	□	□
i. How easy/difficult is it for you to understand warnings about behavior (e.g., smoking, low physical activity, and drinking too much)?	□	□	□	□
j. How easy/difficult is it for you to understand why you need health screenings (such as breast exam, blood sugar, or blood pressure test)?	□	□	□	□
k. How easy/difficult is it for you to judge whether the information on health risks in the media is reliable (e.g., from TV or internet)?	□	□	□	□
l. How easy/difficult is it for you to decide how you can protect yourself from illness based on information in media (e.g., newspapers, leaflets, and internet)?	□	□	□	□
m. How easy/difficult is it for you to find out about activities that are good for your mental well-being (e.g., meditation, exercise, and walking)?	□	□	□	□
n. How easy/difficult is it for you to understand advice on health from your family members or friends?	□	□	□	□
o. How easy/difficult is it for you to understand information in the media on how to get healthier (e.g., from the internet, or daily or weekly magazines)?	□	□	□	□
p. How easy/difficult is it for you to judge which everyday behavior is related to your health (e.g., eating habits, exercise habits, and drinking habits)?	□	□	□	□
